# Prevention of hypertension in patients with pre-hypertension: protocol for the PREVER-prevention trial

**DOI:** 10.1186/1745-6215-12-65

**Published:** 2011-03-05

**Authors:** Flávio D Fuchs, Sandra C Fuchs, Leila B Moreira, Miguel Gus, Antônio C Nóbrega, Carlos E Poli-de-Figueiredo, Décio Mion, Luiz Bortoloto, Fernanda Consolim-Colombo, Fernando Nobre, Eduardo Barbosa Coelho, José F Vilela-Martin, Heitor Moreno, Evandro José Cesarino, Roberto Franco, Andréa Araujo Brandão, Marcos R de Sousa, Antônio Luiz Pinho Ribeiro, Paulo Cesar Jardim, Abrahão Afiune Neto, Luiz César N Scala, Marco Mota, Hilton Chaves, João Guilherme Alves, Dario C Sobral Filho, Ricardo Pereira e Silva, José A Figueiredo Neto, Maria Cláudia Irigoyen, Iran Castro, André Avelino Steffens, Rosane Schlatter, Renato Bandeira de Mello, Francisca Mosele, Flávia Ghizzoni, Otávio Berwanger

**Affiliations:** 1 Hospital de Clínicas de Porto Alegre, Universidade Federal do Rio Grande do Sul, Porto Alegre, Brazil; 2 Hospital Universitário Antônio Pedro, Universidade Federal Fluminense, Niterói, Brazil; 3 Hospital São Lucas, Pontifícia Universidade Católica do Rio Grande do Sul, Porto Alegre, Brazil; 4 Hospital das Clinicas, Universidade de São Paulo, São Paulo, Brazil; 5 Instituto do Coração, Universidade de São Paulo, São Paulo, Brazil; 6 Faculdade de Medicina de Ribeirão Preto - Universidade de São Paulo, Ribeirão Preto, Brazil; 7 Faculdade de Medicina São José do Rio Preto, São José do Rio Preto, Brazil; 8 Faculdade de Ciências Médicas, Universidade de Campinas, Campinas, Brazil; 9 Faculdade de Ciências Farmacêuticas, Universidade de São Paulo, Ribeirão Preto, Brazil; 10 Faculdade de Medicina de Botucatu, Universidade Estadual de São Paulo, Botucatu, Brazil; 11 Universidade do Estado do Rio de Janeiro, Rio de Janeiro, Brazil; 12 Hospital das Clínicas, Universidade Federal de Minas Gerais, Belo Horizonte, Brazil; 13 Hospital das Clínicas de Goiânia, Universidade Federal de Goiás, Goiânia, Brazil; 14 Anis Rassi Hospital, Goiânia, Brazil; 15 Hospital Universitário Júlio Muller, Universidade Federal de Mato Grosso, Cuiabá, Brazil; 16 Faculdade de Medicina, Universidade de Ciências da Saúde Alagoas, Maceió, Brazil; 17 Faculdade de Medicina, Universidade Federal de Pernambuco, Recife, Brazil; 18 Instituto de Medicina Integral Prof Fernando Figueira, Recife, Brazil; 19 Hospital Universitário Oswaldo Cruz/PROCAPE, Universidade de Pernambuco, Recife, Brazil; 20 Hospital Universitário Valter Cantídio, Universidade Federal do Ceará, Fortaleza, Brazil; 21 Hospital Universitário, Universidade Federal Maranhão, São Luiz, Brazil; 22 Instituto de Cardiologia, Fundação Universitária de Cardiologia, Porto Alegre, Brazil; 23 Faculdade de Medicina, Universidade Federal de Pelotas, Pelotas, Brazil; 24 Hospital do Coração, São Paulo, Brazil

## Abstract

**Background:**

Blood pressure (BP) within pre-hypertensive levels confers higher cardiovascular risk and is an intermediate stage for full hypertension, which develops in an annual rate of 7 out of 100 individuals with 40 to 50 years of age. Non-drug interventions to prevent hypertension have had low effectiveness. In individuals with previous cardiovascular disease or diabetes, the use of BP-lowering agents reduces the incidence of major cardiovascular events. In the absence of higher baseline risk, the use of BP agents reduces the incidence of hypertension. The PREVER-prevention trial aims to investigate the efficacy, safety and feasibility of a population-based intervention to prevent the incidence of hypertension and the development of target-organ damage.

**Methods:**

This is a randomized, double-blind, placebo-controlled clinical trial, with participants aged 30 to 70 years, with pre-hypertension. The trial arms will be chlorthalidone 12.5 mg plus amiloride 2.5 mg or identical placebo. The primary outcomes will be the incidence of hypertension, adverse events and development or worsening of microalbuminuria and of left ventricular hypertrophy in the EKG. The secondary outcomes will be fatal or non-fatal cardiovascular events: myocardial infarction, stroke, heart failure, evidence of new sub-clinical atherosclerosis, and sudden death. The study will last 18 months. The sample size was calculated on the basis of an incidence of hypertension of 14% in the control group, a size effect of 40%, power of 85% and P alpha of 5%, resulting in 625 participants per group. The project was approved by the Ethics committee of each participating institution.

**Discussion:**

The early use of blood pressure-lowering drugs, particularly diuretics, which act on the main mechanism of blood pressure rising with age, may prevent cardiovascular events and the incidence of hypertension in individuals with hypertension. If this intervention shows to be effective and safe in a population-based perspective, it could be the basis for an innovative public health program to prevent hypertension in Brazil.

**Trial Registration:**

Clinical Trials NCT00970931.

## Background

High blood pressure is the major risk factor for cardiovascular disease. The risks start at blood pressure values as lower as 115/75 mmHg, but increase exponentially and confer increased absolute risks with blood pressure higher than 140/90 mmHg [1,2]. Cardiovascular disease is already the leading cause of death in Brazil. The prevalence of hypertension in Brazil ranges from 22.3 to 44% of adults [3]. Therefore, interventions aiming to prevent or treat high blood pressure are highly needed. The rationale for precocious drug intervention to prevent hypertension was recently presented [4] and is summarized below.

### Thresholds of risk for blood pressure

A meta-analysis of 61 cohort studies, with more than one million of subjects (12.7 million persons-year at risk), presenting more than 56,000 fatal cardiovascular events, demonstrated that the risk for cardiovascular events starts with systolic blood pressure higher than 115 mmHg or diastolic blood pressure higher than 75 mmHg, doubling at each 20 mmHg in the first case or 10 mmHg in the second [1]. The risks of pre-hypertension and high normal blood pressure have been confirmed in other cohorts [5,6]. The efficacy of blood pressure-lowering drugs to reduce such risks, with a magnitude anticipated by the cohort studies, corroborated in the experimental setting the estimation of risks [7].

### Physiopathological basis for early intervention

The raising of blood pressure with age is not inexorable and does not occur in populations that do not consume large amounts of salt. Under the contemporary and unnatural overload of dietary sodium, kidneys had to reset their primary sodium handling function from retention to excretion. Subjects with familial predisposition to hypertension require higher renal flow and consequently higher blood pressure to eliminate the sodium overload, resulting in extracellular volume expansion, increase in cardiac output and peripheral resistance. With long-standing high blood pressure, loss of glomeruli and renal arterioles may further shift pressure natriuresis and exacerbate blood pressure elevation. The recurrence of this phenomenon along the years leads to arteriolar hypertrophy and sustained blood pressure rise [8-11]. After a long period of high peripheral resistance and diastolic blood pressure, stiffness of large vessels arises, with consequent rise of systolic blood pressure. This deleterious natural history of blood pressure rising with ageing could be aborted in the very beginning by a low-salt diet or by increasing natriuresis, which could be accomplished by very low doses of diuretics or other drugs that enhance the renal capacity of excreting sodium.

### Incidence of hypertension in patients with pre-hypertension

The worldwide prevalence of hypertension in individuals older than 70 years was 70% of women and 59% of men [12]. The incidence of hypertension increases with age until the fifth decade, particularly among individuals with high-normal blood pressure [13]. Four out of five individuals with pre-hypertension aged 40 to 49 years developed hypertension in 10 years in a population-based cohort study conducted in Porto Alegre, Brazil [14]. Therefore, pre-hypertension is not only a risk by itself but it identifies individuals at higher risk for the development of full hypertension in a short period of time.

### Low effectiveness of non-drug interventions in patients with hypertension and pre-hypertension

The risk factors for hypertension, such as the excessive dietary sodium intake, low potassium consumption, excess of adiposity, insulin resistance, abuse of alcoholic beverages, and low consumption of fruits and vegetables are well-known. Many nutritional and behavioral interventions are efficacious to reduce blood pressure, but outside the strict experimental conditions their effectiveness has been inconsistent. The mean weight of populations is not stable but has increased in recent years worldwide. Randomized controlled trials with long follow-up periods have shown that the efficacy of weight control interventions is lost with time. In the TOHP-II trial, weight, salt consumption and blood pressure returned to the baseline values after 36 months of intervention [15]. The DASH diet, which was highly efficacious in a strictly controlled trial [16], was only marginally efficacious in the PREMIER study [17], where differences in blood pressure emerged only when compared with the advice group. The efficacy of low-salt diets was negligible in trials that lasted more than six months [18]. In a clinical context, only the recommendation to lose weight had a short-term effect on blood pressure [19].

### The efficacy of drugs to prevent hypertension in patients with pre-hypertension

Two large clinical trials showed that the prevention of hypertension by drug treatment is feasible and well tolerated. In the TROPHY study [20], 772 individuals with systolic blood pressure between 130 and 139 mmHg or diastolic blood pressure between 85 and 89 mmHg were randomized to candesartan, 16 mg daily or placebo, beside recommendations to change lifestyle. After two years, the incidence of hypertension was 66% lower in individuals treated with candesartan (relative risk 0.34, 95% CI: 0.25-0.44), corresponding to a NNT of 4, which means that four individuals should be treated to prevent one case of hypertension. After two years the treatment was interrupted and blood pressure tended to return to the levels of the control group. The treatment was well tolerated. Similar but not so intense efficacy was observed in the study PHARAO [21], which compared ramipril with placebo.

### Prevention of cardiovascular events in patients with blood pressure within normal values

The expectation that BP agents have beneficial effects independent of the blood pressure-lowering one (pleiotropic effects) is still deep-rooted in physician's minds, but it has been repeatedly dismissed by independent clinical trials and by a large meta-analysis [7] The efficacy of beta-blockers in patients recovering from a myocardial infarction and in patients with heart failure, of ACE inhibitors in patients with heart failure, coronary heart disease, other evidences of atherosclerotic vascular disease or diabetes, and patients recovered from a stroke (with a diuretic) can be almost fully explained by the blood pressure-lowering effect [22]. At least part of the evidences supporting the existence of such effects may have resulted from biases in the planning and interpretation of clinical trials funded by pharmaceutical companies [23].

### Trial rationale

The early use of blood pressure-lowering drugs may prevent cardiovascular events and the incidence of hypertension. The very low absolute risk of pre-hypertension in young individuals free of diabetes or cardiovascular disease precludes the launching of clinical trials with the aim to prevent hard outcomes. The blood pressure-lowering effect and the prevention of the incidence of full hypertension may be valid surrogate endpoints that could be investigated in feasible randomized clinical trials. The PREVER prevention trial study will test if a low dose of an association of chlorthalidone with amiloride prevents hypertension at an acceptable safety in a nationwide large sample of individuals with pre-hypertension (Clinical trials registration number: NCT00970931). Other intermediate outcomes, such as microalbuminuria and left ventricular hypertrophy, are going to be investigated. Diuretics were chosen in face of their cost-effectiveness, since they are at least as efficacious as other blood pressure agents [24], at very low frequency of adverse effects, and the fact that they are the cheapest agents. Chlorthalidone was chosen in face of its higher blood pressure-lowering efficacy and better performance in the prevention of major cardiovascular events than hydrochlorothiazide [24]. The association with amiloride aims to prevent the deleterious hypokalemia induced by diuretics [25,26]. If the intervention shows to be effective and well-tolerated, the early use of this association could be the basis for an innovative public health program to prevent hypertension in Brazil.

### Research questions

1. Does an association of low doses of chlorthalidone and amiloride reduce the incidence of hypertension in individuals with pre-hypertension?

2. Does an association of low doses of chlorthalidone and amiloride reduce the incidence of target-organ damage in patients with pre-hypertension?

3. Does an association of low doses of chlorthalidone and amiloride reduce the incidence of cardiovascular events in patients with pre-hypertension?

4. Is the association of low doses of chlorthalidone and amiloride safe to be used on a population-based perspective?

## Methods

### Design

randomized, double-blind, clinical trial, controlled by placebo.

### Eligible participants

individuals with 30 to 70 years of age with pre-hypertension.

### Exclusion criteria

low life expectancy, other indications for the use of diuretics, such as cardiovascular disease, intolerance to the study drugs, pregnancy.

### Random allocation

will be generated by a computer, using a validated software (Random allocator), with variable block sizes and stratified by center.

### Interventions

**Figure 1:**
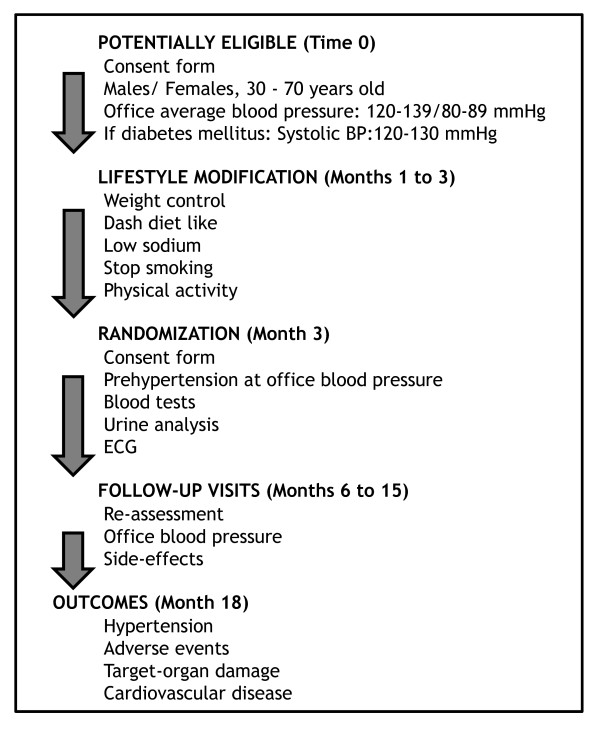
**Flow chart of the PREVER-prevention trial, describing selection, randomization and follow-up process**.

Chlorthalidone 12.5 mg plus amiloride 2.5 mg or identical placebo. Figure 1 shows flow-chart of selection of participants and interventions.

### Outcomes

Primary:

1. Incidence of hypertension.

2. Adverse events.

3. Development or worsening of microalbuminuria and of left ventricular hypertrophy in the EKG.

Secondary: fatal or non-fatal cardiovascular events.

### Follow-up and duration of the study

**Figure 2:**
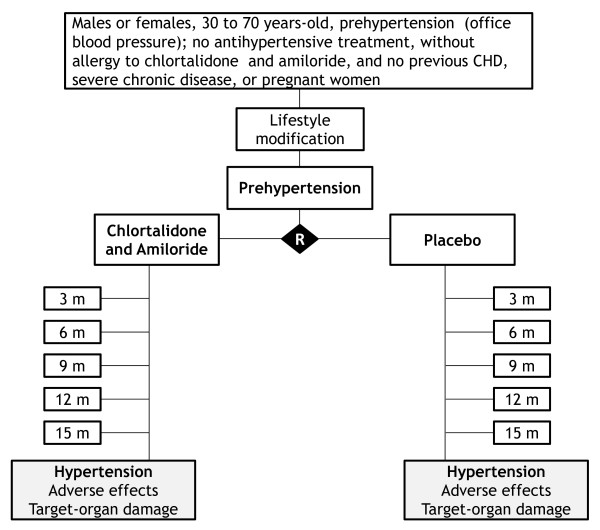
**Summary of the PREVER-Prevention trial key practical aspects**.

consultations for evaluation and enrollment and thereafter consultations at the third, 6^th^, 9^th^, 12^th ^and 18^th ^months. Figure 2 shows the summary of key practical aspects of the trial. Lifestyle interventions will be applied before the randomization and will be maintained throughout the trial. An extended follow-up is planned pending on additional funding.

### Assessment of outcomes

Incidence of hypertension: the diagnosis of hypertension will be signaled at any of the follow-up visits by average blood pressure (two measurements by an automatic electronic device Microlife BP 3BTO-A, licensed for fabrication by Micromed Biotecnologia Ltda, Brasília, Brazil) ≥ 140/90 mmHg. The diagnosis will be confirmed by a new visit soon afterwards, with the average of blood pressure measurements of both consultations within hypertensive levels.

Adverse events: adverse events will be investigated by open questions and by a semi-structured questionnaire including general symptoms and the presumed adverse effects of the drugs used in the trial. The incidence of adverse effects will be determined by the incidence of adverse events more frequent in the drug arm than in the placebo arm. Laboratory adverse events, such as hypokalemia, hyperuricemia and diabetes (glycated hemoglobin and fasting glucose) will be investigated at the final visit of the participants.

Target-organ damage: Microalbuminuria will be determined by nephelometry. Left ventricular hypertrophy will be investigated by electrocardiogram, using the Sokolow-Lyon voltage and the Cornell voltage-duration product. These examinations will be carried out at the baseline evaluation and the final visit.

**Figure 3:**
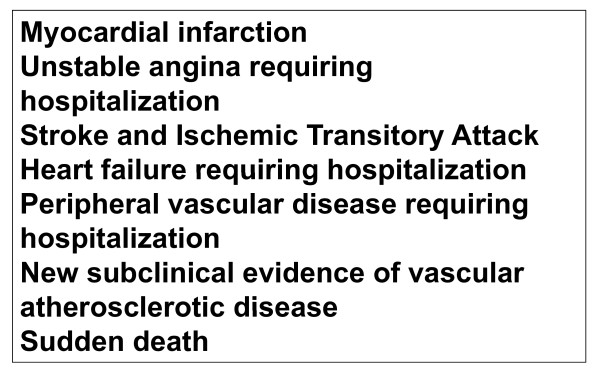
**PREVER-prevention trial cardiovascular outcomes**.

Cardiovascular outcomes: from a statistical point of view they will be secondary events, in face of the sample power calculated for the trial. Figure 3 presents the main outcomes that will be investigated. The cases will be adjudicated on the basis of interview, hospital charts and exams, death certificates and verbal autopsy with next-of-kin, by members of the outcome committee.

### Sample size calculation

assuming an incidence of hypertension of 14% in two years in the control group, a size effect of 40% of reduction in the incidence in the treated group, for a power of 85% and P alpha of 5%, it will be necessary to study 568 individuals per group, rounded to 625 per group to compensate for losses in follow-up.

### Statistics

the cumulative incidence of hypertension and adverse events will be tested by Chi-square. Survival curves (Kaplan Meyer) will be compared by log-rank test, and Cox hazard regression models. Blood pressure will be compared by ANOVA for multiple repeated measurements and factors, testing for the time-group interaction.

### Logistics

this is a nation-based trial, with 24 clinical centers distributed in nine States. A Coordinating Committee was responsible for the elaboration of this proposal and for the main decisions regarding the trial. The organizational study chart will include an executive Committee, a safety committee, outcome committee, data, lab and EKG centers, and the research units

## Discussion

The early use of blood pressure-lowering drugs in individuals with pre-hypertension may prevent cardiovascular events, target-organ damage and the incidence of full hypertension. Non-drug treatment (life-style changing) has had low long-term effectiveness and blood pressure drugs can circumvent such limitation. Diuretics may be particularly efficacious in this regard, since they act on the main mechanism of blood pressure rising with ageing. The association of chlorthalidone with amiloride combines the efficacy of chlorthalidone with the potassium-sparing effect of amiloride, preventing electrolyte and metabolic abnormalities induced by chlorthalidone. If this association shows to be effective and well-tolerated on a population-based perspective, it could be the basis for an innovative public health program to prevent hypertension in Brazil.

### Grants

the Ministry of Health, Division of Science and Technology (DECIT), and Ministry of Science and Technology, FINEP and CNPq, Brazil, funded the study.

### Ethical approval

The project was approved by the Ethics committee of the Hospital de Clínicas de Porto Alegre, which is accredited by the Office of Human Research Protections as an Institutional Review Board, and by the Ethic Committees of all participating centers. All participants will sign an informed consent to participate.

## Competing interests

The authors declare that they have no competing interests.

## Authors' contributions

FDF conceived the study; all authors participated in the trial design and methodological considerations, contributed to the draft of this manuscript for intellectual content and approved its final version. They are the coordinators of the clinical centers that will enroll the trial participants.

## List of abbreviations

BP: blood pressure; NNT: number needed to treat; ACE: Angiotensin converting inhibitors; EKG: Electrocardiogram.
